# Carrier multiplication in silicon nanocrystals: ab initio results

**DOI:** 10.3762/bjnano.6.33

**Published:** 2015-02-02

**Authors:** Ivan Marri, Marco Govoni, Stefano Ossicini

**Affiliations:** 1 Department of Science and Methods for Engineering (DISMI), via Amendola 2, Pad. Morselli, 42122 Reggio Emilia, Italy; 2 Department of Physics, University of Modena and Reggio Emilia, via Campi 213/a, 41125 Modena, Italy; 3 present address: Institute for Molecular Engineering, The University of Chicago, 5555 South Ellis Avenue, Chicago, Illinois 60637, United States

**Keywords:** carrier multiplication, nanocrystals, silicon, solar cells

## Abstract

One of the most important goals in the field of renewable energy is the development of original solar cell schemes employing new materials to overcome the performance limitations of traditional solar cell devices. Among such innovative materials, nanostructures have emerged as an important class of materials that can be used to realize efficient photovoltaic devices. When these systems are implemented into solar cells, new effects can be exploited to maximize the harvest of solar radiation and to minimize the loss factors. In this context, carrier multiplication seems one promising way to minimize the effects induced by thermalization loss processes thereby significantly increasing the solar cell power conversion. In this work we analyze and quantify different types of carrier multiplication decay dynamics by analyzing systems of isolated and coupled silicon nanocrystals. The effects on carrier multiplication dynamics by energy and charge transfer processes are also discussed.

## Introduction

An important challenge in modern day scientific research is the establishment of clean, inexpensive, renewable energy sources. Based on the extraction of energy from the solar spectrum, photovoltaics (PV) is one of the most appealing and promising technologies in this regard. Intense effort is focused on increasing solar cell performance through the minimization of loss factors and the maximization of solar radiation harvesting. This is accomplished by improving the optoelectronic properties of existing devices and by realizing new schemes for innovative solar cell systems. For optimal energy conversion in PV devices, one important requirement is that the full energy of the solar spectrum is used. In this context, the development of third generation nanostructured solar cells appears as a promising way to realize new systems that can overcome the limitations of traditional, single junction PV devices. The possibility of exploiting features that derive from the reduced dimensionality of the nanocrystalline phase, and in particular, features induced by the quantum confinement effect [1–5] can lead to a better use of the carrier excess energy, and can increase solar cell thermodynamic conversion efficiency over the Shockley–Queisser (SQ) limit [6]. In this context, carrier multiplication (CM) can be exploited to maximize solar cell performance, promoting a net reduction of loss mechanisms. CM is a Coulomb-driven, recombination process that occurs when a highly excited carrier (excess energy of the excited carrier is higher than the band gap energy, *E*_g_) decays to a lower energy state by transferring its excess energy to generate extra e–h pairs. When CM involves states of the same nanostructure, the effect is termed one-site CM. Because of the restrictions imposed by energy and momentum conservation and by fast phonon relaxation processes, CM is often inefficient in bulk semiconductors. On the nanoscale, CM is favored (a) by quantum confinement that enhances the carrier–carrier Coulomb interaction [7], (b) by the lack of restrictions imposed by the conservation of momentum [8] and, in some cases, (c) by the so-called “phonon bottleneck” effect [9–10] that reduces the probability of exciton relaxation by phonon emission. These conditions make the formation of multiple e–h pairs after absorption of high energy photons more likely to occur in low-dimensional nanostructures. Consequently, at the nanoscale CM can be as fast as (or faster than) phonon scattering processes and Auger cooling mechanisms [11]. Therefore, CM represents an effective way to minimize energy loss factors and constitutes a possible route for increasing solar cell photocurrent, and hence, to increase solar cell efficiency. Effects induced by CM on the excited carrier dynamics have been observed in a wide range of systems, for instance PbSe and PbS [12–16], CdSe and CdTe [17–19], PbTe [20], InAs [21], InP [22] and Si [23]. These effects have been studied using different theoretical approaches [21,24–30] although only recently was a full ab initio interpretation of CM proposed [31]. Recently, a relevant photocurrent enhancement arising from CM was observed in a PbSe-based, quantum dot (QD) solar cell [32], which proves the possibility of exploiting CM effects to improve solar cell performance. In this context, the possibility to use the non-toxic and largely diffused silicon instead of lead-based materials can be advantageous to the future development of QD-based solar cell devices. A new CM scheme was recently hypothesized by Timmerman et al. [33–35] and by Trinh et al. [36] in order to explain results obtained in photoluminescence (PL) and induced absorption (IA) experiments conducted on dense arrays of silicon nanocrystals (Si-NCs, NC–NC separation ≤ 1 nm). In the first set of experiments, the authors proved that although the excitation cross-section is wavelength-dependent and increases for shorter excitation wavelengths, the maximum time-integrated PL signal for a given sample saturates at the same level independent of the excitation wavelength or the number of generated e–h pairs per NC after a laser pulse. In this case, saturation occurs when every NC absorbs at least one photon. This process was explained by considering a new energy transfer-based CM scheme, space-separated quantum cutting (SSQC). CM by SSQC is driven by the Coulomb interaction between carriers of different NCs and differs from traditional CM dynamics because the generated e–h pairs are localized onto different interacting NCs. By distributing the excitation among several nanostructures, CM by SSQC represents one of the most suitable routes for solar cell loss minimization. Subsequent experiments conducted by Trinh et al. [36] pointed out the lack of fast decay components in the IA dynamics for high energy excitations (*h*ν > 2*E*_g_). For such photoexcitation events, the intensity of the IA signal was proven to be twice that recorded at an energy below the CM threshold (*h*ν ≈ 1.6*E*_g_); this argument was used to prove the occurrence of CM effects in dense arrays of Si-NCs. Experimental results were interpreted by hypothesizing a direct formation of e–h pairs localized onto different NCs by SSQC. The measured quantum yield was proven to be very similar to that measured in the PL experiments conducted by Timmerman et al. [33–35], pointing to a similar microscopic origin of the recorded PL and IA signals.

In this work, we investigate effects induced on CM dynamics using first principles calculations. One-site CM, Coulomb-driven charge transfer (CDCT) and SSQC processes are evaluated in detail and a hierarchy of CM lifetimes are noted.

## Theory

In this work we investigate CM effects in systems of isolated and interacting Si-NCs. Structural and electronic properties are calculated within the density functional theory (DFT) using the local density approximation, as implemented in the QuantumESPRESSO package [37]. Energy levels are determined by considering a wavefunction cutoff of 20 Hartree. Following Rabani et al. [29], CM rates are calculated by applying first order perturbation theory (Fermi’s golden rule, impact ionization decay mechanism) by separating processes ignited by electrons (h spectator) and holes (e spectator), that is:

[1]
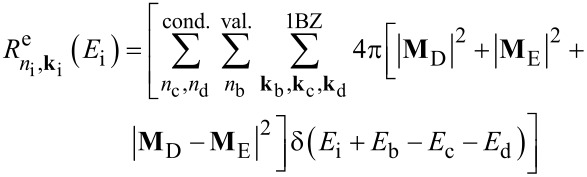


and

**Figure 1:**
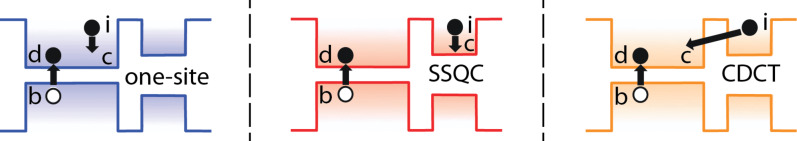
A schematic representation of one-site CM, SSQC and CDCT (for more details see [41]). When SSQC occurs, a highly excited carrier decays to lower energy states, transferring its excess energy to a close NC where an extra e–h pair is generated.

[2]
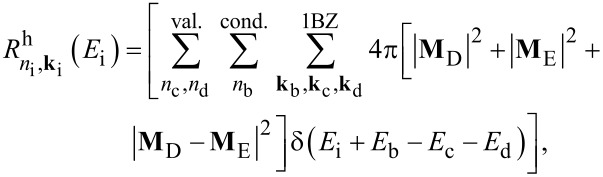


where the superscripts “e” and “h” identify mechanisms ignited by relaxation of an electron and a hole, respectively. In Equation 1 and Equation 2, the rates are expressed as a function of the energy of the initial carrier, without considering the lattice vibration (a detailed ab initio calculation of phonon-assisted CM processes currently represents, for the considered systems, an unattainable task that goes beyond the scope of this work). The label *n*_i_**k**_i_ denotes the Kohn–Sham (KS) state of the carrier that ignites the transition, while *n*_b_**k**_b_, *n*_c_**k**_c_ and *n*_d_**k**_d_ identify the final states (see Figure 1). **M**_D_ and **M**_E_ are the two particle direct and exchange Coulomb matrix elements [38] calculated between KS states. Energy conservation is imposed by the presence of the delta function (it is implemented in the form of a Gaussian distribution with a full width at half maximum of 0.02 eV). The screened Coulomb potential, which is the basis of the calculation of both **M**_D_ and **M**_E_, is obtained by solving Dyson’s equation in the random phase approximation, as implemented in the many-body YAMBO code [39]. In reciprocal space, the Fourier transform of the zero-frequency screened Coulomb potential is given by:

[3]
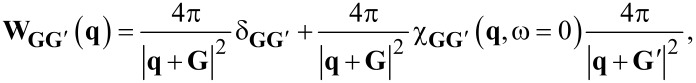


where **G** and **G**’ are vectors of the reciprocal lattice, **q** *=* (**k**_c_ − **k**_i_)_1BZ_, and χ**_GG'_** is the reducible, zero frequency, density–density response function. The first term on the right-hand side of Equation 3 represents the bare part of the Coulomb potential, and the second term defines the screened part. The presence of off-diagonal elements in the solution of Dyson’s equation is related to the inclusion of local fields. CM lifetimes are then obtained as a reciprocal of rates, that is

[4]
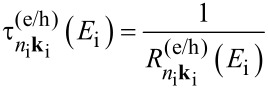


found by calculating the inverse of the sum of all CM rates able to connect the initial *n*_i_**k**_i_ state with the final states, satisfying the energy conservation law within 0.05 eV. Spurious Coulomb interactions among nearby replicas are avoided thanks to the use of the box-shaped, exact cutoff technique [40].

When two NCs are placed in close proximity, wavefunctions are able to delocalize on the entire system and new CM effects emerge from NC–NC interaction. In this condition, the total CM rate can be split in two parts: (a) one-site CM processes, where initial and final states are localized onto the same NC and (b) two-site CM effects, where initial and final states are localized onto different NCs, that is, SSQC and CDCT. SSQC is a Coulomb-driven, energy transfer process that occurs when a high energy electron (hole) decays toward the conduction (valence) band CB (VB) edge by promoting the formation of an extra e–h pair in a nearby NC. CDCT, instead, is a Coulomb-driven, charge transfer mechanism that occurs when an electron (hole) decays toward the CB (VB) of a nearby NC where an extra e–h pair is generated (see Figure 1).

One of the simplest way to represent a system of interacting NCs is to place two different NCs in the same simulation box, at a tunable separation, *d*. In our work, the largest NC is placed in the left part of the box while the smaller NC is placed into the right part of the cell. The NCs are equidistant with respect the center of the cell. In order to quantify both the one-site and two-site CM lifetimes, we introduce a new parameter, the spill-out parameter 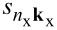, which defines the localization of a specific KS state *n*_x_**k**_x_ onto the smaller NC. This parameter is obtained by integrating the wavefunction square modulus 
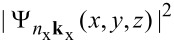
 over the volume of the cell that is occupied by the smaller NC, that is:


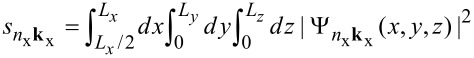


where *L_x_*, *L_y_* and *L_z_* are the box cell edges. When the electronic state 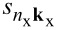 is completely localized on the smallest (largest) NC then 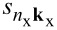 = 1 (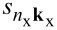 = 0). Otherwise, when the state *n*_x_**k**_x_ is spread over both NCs, then 0 < 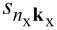 < 1. For a system of interacting NCs, the one-site CM rate is given by

[5]
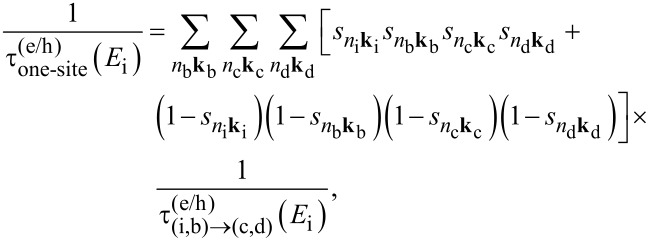


where 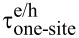 (*E*_i_) is the one-site CM lifetime for a process ignited by a carrier of energy *E*_i_. 
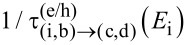
 is the total CM rate for the generic, single, CM decay path (i,b) → (c,d) (see Figure 1).


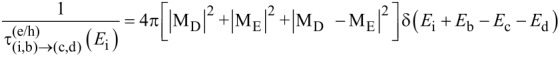


and the weighting factors 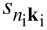, 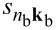, 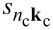, and 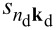 are the spill-out parameters of the states *i*, *b*, *c* and *d*.

Equation 5 is obtained by weighting the single CM rate 
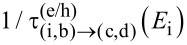
 of a permitted CM decay path (i,b) → (c,d) with the product of the spill-out parameters and by summing over all possible final states [42]. At the same time the SSQC rate is obtained by considering the portion of the wavefunctions of the states i and c that are localized onto the smallest (largest) NC and the portion of the states b and d that are localized onto the largest (smallest) NC, that is:

[6]
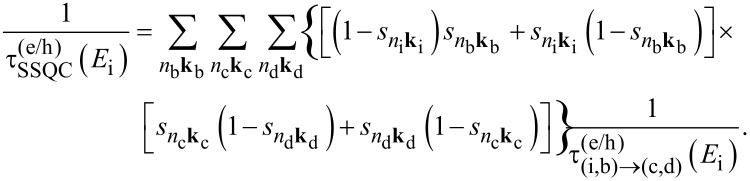


The CDCT rate can be trivially obtained by:

[7]
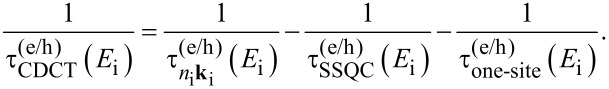


In our work, we consider four different isolated Si-NCs: (Si_35_H_36_, Si_87_H_76_, Si_147_H_100_ and Si_293_H_172_), and a couple of interacting NCs (Si_87_H_76_ × Si_293_H_172_). For all of the systems considered, the NCs are always assumed in vacuum.

## Results and Discussion

CM effects in isolated and interacting Si-NCs were investigated for the first time by first-principles calculations by Govoni et al. [31], who simulated CM decays in systems of isolated and interacting Si-NCs. CM lifetimes were calculated in four different spherical and hydrogenated systems, that is the Si_35_H_36_ (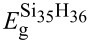 = 3.42 eV, 1.3 nm of diameter), the Si_87_H_76_ (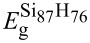 = 2.50 eV, 1.6 nm diameter), the Si_147_H_100_ (
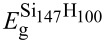
 = 2.21 eV, 1.9 nm diameter) and the Si_293_H_172_ (
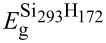
 = 1.70 eV, 2.4 nm diameter).

Systems of strongly coupled Si-NCs (Si_35_H_36_ × Si_293_H_172_ and Si_147_H_100_ × Si_293_H_172_) were then analyzed in order to define effects induced by NC interplay on CM effects.

In this work we investigate new aspects of CM dynamics in both isolated and interacting Si-NCs. For the first step, we reconsider the systems Si_35_H_36_, Si_87_H_76_, Si_147_H_100_ and Si_293_H_172_ and we analyze the dependence of CM lifetimes on NCs size. The role played by local fields (and in general by the screened part of the Coulomb potential) on CM dynamics is successively analyzed. The system of strongly coupled NCs (Si_87_H_76_ × Si_293_H_172_) was then studied to investigate effects induced by NC interplay on CM decay processes. The resulting CM lifetimes are then compared with those obtained in [31] for the systems Si_35_H_36_ × Si_293_H_172_ and Si_147_H_100_ × Si_293_H_172_ in order to investigate the dependence of the two-site CM effect on NC size. The role played by reciprocal NCs orientation is finally briefly analyzed.

**Figure 2:**
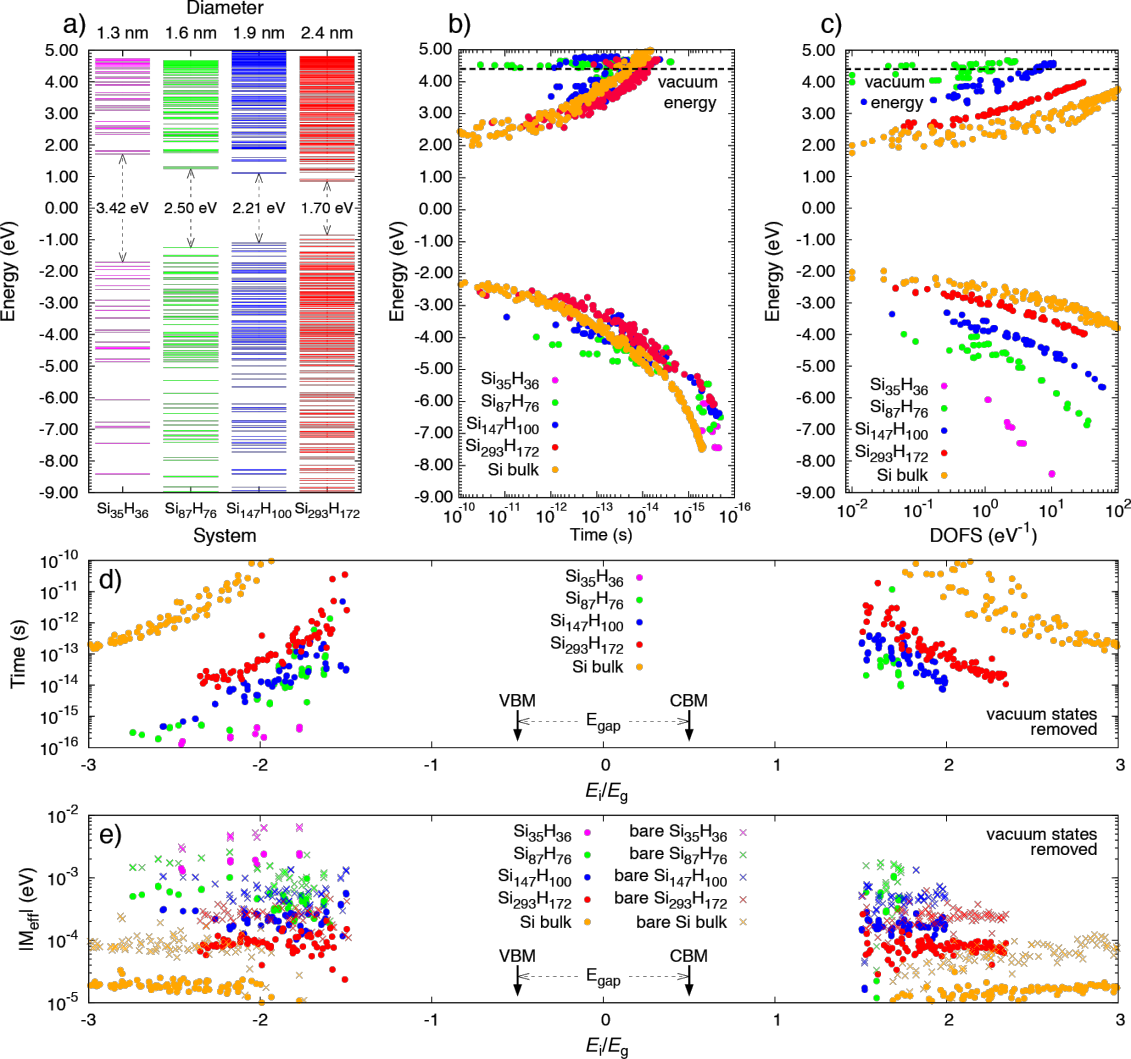
Electronic structures of Si_35_H_36_, Si_87_H_76_, Si_147_H_100_ and Si_293_H_172_ are reported in (a). CM lifetimes calculated for the considered Si-NC systems and for the Si-bulk are reported in (b) and (d). Both mechanisms which are ignited by electron relaxation (positive energy) and hole relaxation (negative energy) are considered. In (b), CM lifetimes are given as a function of the energy of the the initial carrier, *E*_i_. In (d) CM lifetimes are expressed in terms of the ratio *E*_i_/*E*_g_. The zero of the energy scale is set at the half band gap for each NC system. Dashed horizontal lines in (b) and (c) denote the vacuum energy level. In our calculations, we omit vacuum states, that is, conduction band states with an energy higher than the vacuum energy. The calculated density of final states are reported in (c). The results were obtained considering a broadening of 5 meV. The effective Coulomb matrix elements are given in (e). The filled circle data points represent results obtained by including both bare and screened terms in Equation 3 and colored crosses represent only the bare terms of Equation 3.

CM lifetimes calculated for the isolated Si-NC systems are reported in Figure 2 as a function of both the energy of the initial carrier ((b) absolute energy scale) and the ratio between the energy of the initial carrier and the energy gap of the system (*E*_i_/*E*_g_, (d), relative energy scale). In both cases, CM lifetimes are obtained by omitting vacuum states, which are conduction levels above the vacuum energy.The calculated CM lifetimes for Si-NCs are then compared with those obtained for Si-bulk (yellow points). The results of Figure 2 indicate that CM is forbidden when the excess energy, *E*^exc^, of the initial carrier is lower than *E*_g_. On the contrary, when |*E*^exc^| > |*E*_g_|, CM is permitted and the calculated CM lifetime, after initial fluctuations, decreases when the energy of the initial carrier increases. When an absolute energy scale is adopted (Figure 2b) and low energy dynamics are analyzed, CM is strongly influenced by the energy gap of the system and is faster in systems with lower *E*_g_, that is, the Si-bulk (energy range of approximately −2.5 eV < *E*_i_ < 2.5 eV). However, under these conditions, CM is generally not sufficiently fast to dominate over concurrent decay mechanisms and can only weakly affect the time evolution of the excited carrier. For Si-NCs, thermalization processes are expected to range from a few picoseconds to a fraction of a picosecond [43–44]. In the ranges −3.8 eV < *E*_i_ < −2.5 eV and 2.5 eV < *E*_i_ < 3.8 eV, the CM lifetimes calculated for the Si_293_H_172_ are lower than those obtained for the Si-bulk. For the remainder of the plot, that is, approximately for *E*_i_ < −3.8 eV and *E*_i_ > 3.8 eV, CM is faster in Si-NC systems than in Si-bulk and is observed to be independent of the NC size. In this range of energies, CM is sufficiently fast to compete with concurrent non-CM processes and, playing a fundamental role in the determination of the excited carrier dynamics, can be exploited to improve solar cell performance. Analysis of high energy, CM decay paths is therefore fundamental and can have a strong impact on the engineering of new PV devices. The behavior recorded at high energies (where CM lifetimes are independent of the NC size) can be interpreted by reformulating Equation 1 and Equation 2 in order to point out the dependence of the CM rate on the density of final states. Following Allan et al. [24]:

[8]
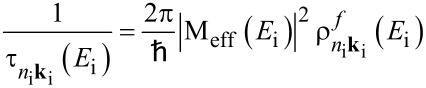


where |*M*_eff_(*E*_i_)| is the effective two-particle, Coulomb matrix element and 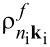
(*E*_i_) is the density of final states. Calculations of 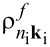
(*E*_i_) *and |M*_eff_(*E*_i_)| are reported in Figure 2c and Figure 2e for both Si-NCs and Si-bulk (Coulomb matrix elements are calculated for both by including and neglecting the screened term, indicated by the dot-type and cross-type points, respectively, of Figure 2e). Our results indicate that, while the effective Coulomb matrix elements (and therefore their squared modulus) decrease with increasing NC size, the density of final states increases with increasing NC size. Far from the activation threshold (approximately −3.8 eV < *E*_i_ and *E*_i_ > 3.8 eV) we observe a sort of exact compensation between the trends of |*M*_eff_(*E*_i_) |^2^ and of 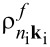
(*E*_i_) that make 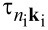
(*E*_i_) almost NC-size-independent. Again, from Figure 2e, we observe that due to the strong discretization of NC electronic states near the VB and CB, the effective Coulomb matrix elements scatter among different orders of magnitude when they are calculated at energies near the CM thresholds. Such oscillations strongly affect the CM lifetimes at low energies and generate fluctuations that are clearly visible in both the plots of Figure 2b and Figure 2d. Instead, at high energies, the effective Coulomb matrix elements stabilize at constant values that depend only on the NC size. Therefore, in this portion of the energy range, the typical trend of 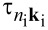
(*E*_i_), which decreases when the energy of the initial state increases, is only ascribable to the monotonically increasing behavior of 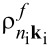
(*E*_i_).

A realistic estimation of CM lifetimes requires a detailed evaluation of the carrier–carrier Coulomb interaction. Due to the required computational and theoretical efforts necessary to solve Equation 3, the Coulomb potential is often approximated by considering only the bare term. The inclusion of the screened part of the Coulomb potential, which requires a detailed estimation of the many-body interacting polarizability, is often neglected in order to make the procedure that leads to the calculation of the dielectric function more manageable. In order to quantify the role played by the screened part of the Coulomb potential, we calculate effective Coulomb matrix elements by adopting two different procedures: firstly, by omitting and then, by including the second term on the right-hand side of Equation 3. The results of Figure 2e illustrate that the inclusion of the screened part of the Coulomb potential leads to effective Coulomb matrix elements that are up to one-order of magnitude smaller that those obtainable by only considering the bare Coulomb interaction. As a consequence, a simplified procedure that avoids the complete calculation of Equation 3 (and therefore also neglects the inclusion of local field effects) leads to an overestimate of the efficiency of CM decay mechanisms and does not allow for a realistic determination of high energy, excited carrier dynamics. It is thus evident that a detailed estimation of 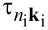
(*E*_i_) requires an accurate description of the atomistic properties of the systems that, especially for nanostructures, can be obtained only through a parameter-free, ab initio investigation of the electronic properties of the considered materials.

A clear dependence of CM lifetimes on NC size appears when a relative energy scale is adopted (plot of Figure 2d), that is, when the CM lifetimes are related to *E*_i_/*E*_g_. As proven by Beard et al. [45], this scale is the most appropriate to predict the possible implication of the CM for PV applications. Thus, from this perspective, there are clear advantages which are induced by size reduction, that is, when moving from the Si-bulk scale to the nanoscale for Si_35_H_36_, as supported by results of Figure 2d.

**Figure 3:**
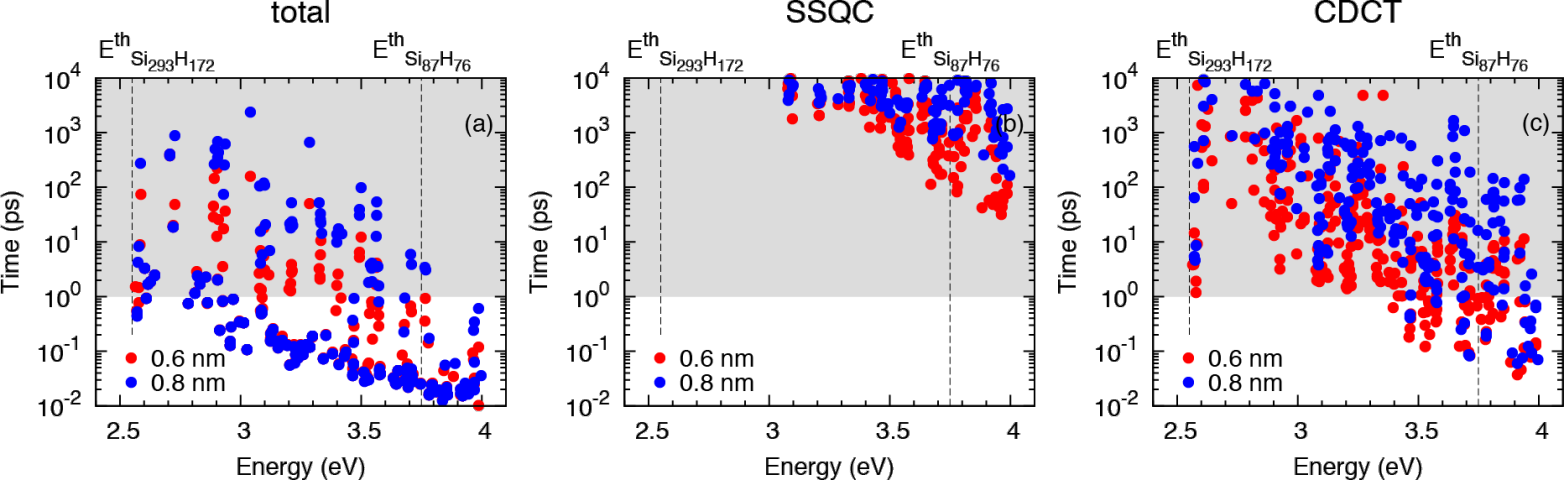
Calculated total CM, SSQC and CDCT lifetimes are reported in (a), (b) and (c), respectively, for the system Si_87_H_76_ × Si_293_H_172_, where NC–NC separations of 0.8 and 0.6 nm (blue and red points, respectively) are given. 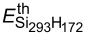 and 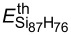 denote the CM energy threshold of the isolated NCs, that is for the Si_293_H_172_ and the Si_87_H_76_ NCs.

In order to study the effects induced by NCs on the interplay of CM dynamics, we consider the system Si_87_H_76_ × Si_293_H_172_ that is obtained by placing in the same simulation box (box size 9.0 × 4.8 × 4.8 nm) two different NCs placed at a tunable separation. As illustrated in Equation 5, Equation 6 and Equation 7, the wavefunction delocalization plays a fundamental role in the determination of one-site CM, CDCT and SSQC lifetimes when systems of strongly interacting NCs are considered. As discussed in [31], the wavefunction delocalization processes (and the effects induced by them) become relevant for NC–NC separations of *d* ≤ 1.0 nm. As a consequence, we analyze the effects induced by NC interplay on CM decay processes by only considering NC–NC separations that fall in the sub-nm regime, and in particular by assuming *d* = 0.8 nm and *d* = 0.6 nm. In our work, the NC–NC separation is the distance between the nearest Si atoms that are localized on different NCs. The calculated CM lifetimes obtained by summing the contributions of Equation 5, Equation 6 and Equation 7 are reported in Figure 3a as a function of the energy of the initial carrier and of the NC–NC separation, *d* (total CM lifetimes).

The calculated SSQC and CDCT lifetimes (mathematically characterized by Equation 6 and Equation 7) are depicted in Figure 3b and Figure 3c. Only mechanisms ignited by electron relaxation are considered. The analysis of the results of Figure 3 leads to the conclusions which are outlined in the following.

First, by changing the separation from *d* = 0.8 to *d* = 0.6 nm, some changes emerge in the plot of the CM lifetimes (Figure 3a). As a result of the improved NC–NC interaction, we observe the drift of some points toward reduced lifetimes. Such changes essentially concern the portion of the plot delimited by the energies 
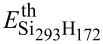
 and 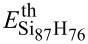 (i.e., the CM energy threshold of the isolated NCs) and by the lifetimes of 1–100 ps. At *d* = 0.6 nm, the distribution of the points is less scattered than for *d* =0.8 nm and moves toward that of an isolated, unique, large system (a similar behavior also characterizes the system Si_147_H_100_ × Si_293_H_172_, see [41]).

Additionally, NC interplay does not significantly alter the faster CM decay processes. This conclusion can be obtained by analyzing the region of Figure 3a that takes into account the CM relaxation mechanisms with a lifetime less than 0.1 ps. Here we observe that blue (*d* = 0.8 nm) and red (*d* = 0.6 nm) points are almost identical. The number of CM decay paths recorded in this region of the plot does not improve when we move from *d* = 0.8 nm to *d* = 0.6 nm.

When the NC–NC separation is reduced, the NC interplay increases, and two-site CM mechanisms become fast. At high energy, τ_CDCT_ ranges from tens of ps to a fraction of a ps, while τ_SSQC_ ranges from hundreds of picoseconds to a few tens of picoseconds. Both the CDCT and SSCQ lifetimes decrease when the NC separation decreases, as a consequence of both the augmented Coulomb interaction between carriers of different NCs and the increased delocalization of wavefunctions.

Another conclusion reached is that CDCT processes are in general faster than SSQC mechanisms. In order to be efficient, CDCT requires a noticeable delocalization of only the initial state while SSQC requires a significant delocalization of all the states involved in the transition; as a consequence, the CDCT decay processes are in general favored with respect to the corresponding SSQC mechanisms.

Finally, despite the fact that NC interplay can enhance the two-site CM processes, the Si_87_H_76_ × Si_293_H_172_ satisfies the typical hierarchy of lifetimes τ_one−site_ ≤ τ_CDCT_ ≤ τ_SSQC_ expected. As a consequence, the system Si_87_H_76_ × Si_293_H_172_ also follows this recently identified trend for the Si_35_H_36_ × Si_293_H_172_ and the Si_147_H_100_ × Si_293_H_172_ systems. Thus, for a given energy of the initial state, one-site CM mechanisms result faster than CDCT processes, and CDCT processes result faster than SSQC mechanisms.

Remarkably, the relevance of the two-site CM processes are expected to benefit from experimental conditions where the formation of minibands (the presence of molecular chains that interconnect different NCs and for multiple interacting NCs) amplify the importance of both the energy and charge CM dynamics. Again, by comparing the results of Figure 3b with the corresponding CM lifetimes calculated in [31], we can say that the efficiency of SSQC processes tends to increase with increasing NC size. In general, experiments are conducted on nanostructured systems that are larger than those considered in this work. As a consequence, in a realistic system, both SSQC and CDCT dynamics could be faster than those computed herein, although these effects should not give rise to changes in the previously discussed hierarchy of lifetimes. The CM is driven by Coulomb interaction and therefore its relevance is maximized when the effect involves carriers localized onto the same NC.

**Figure 4:**
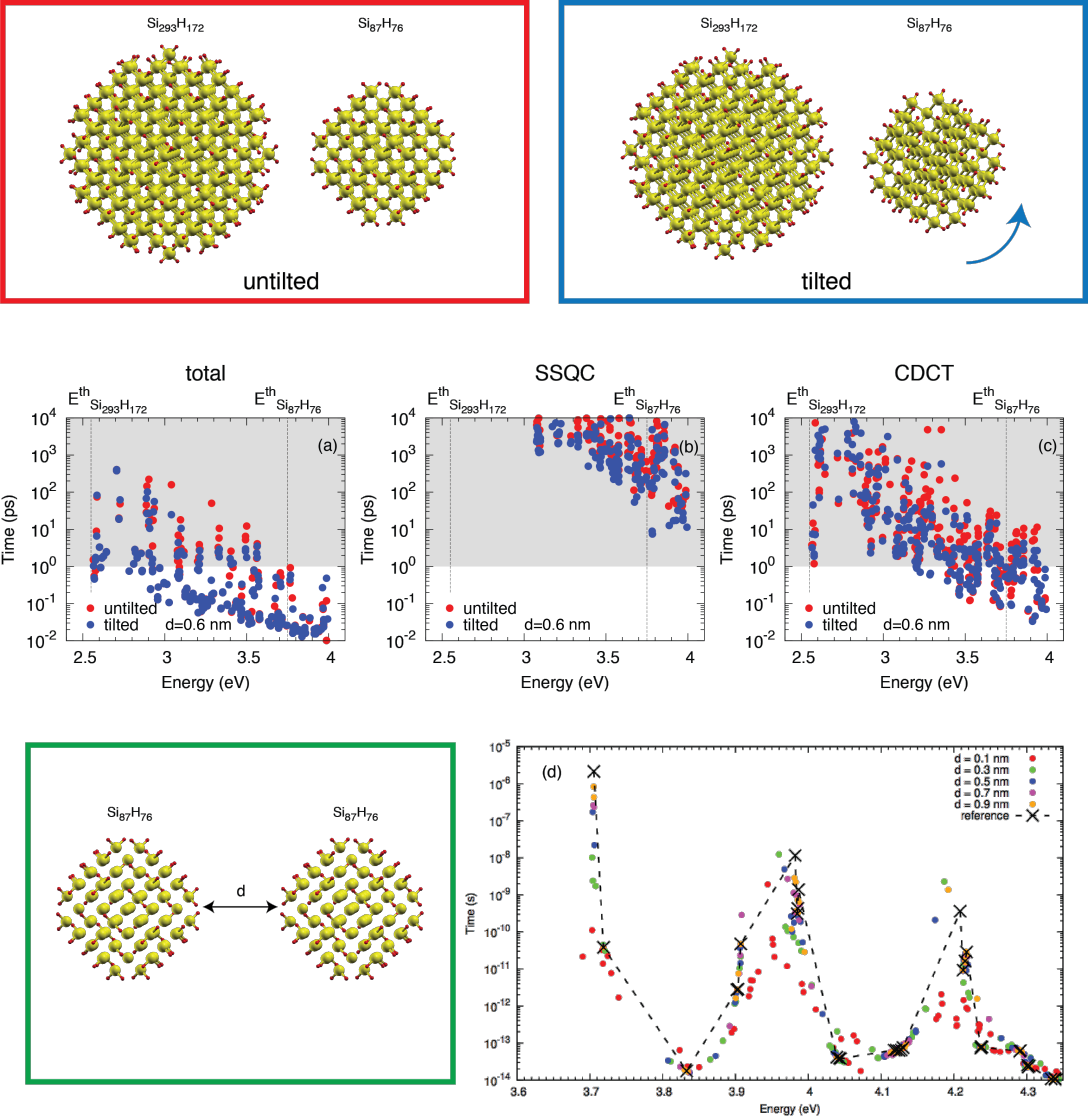
A representation of the systems Si_87_H_76_ × Si_293_H_172_ and 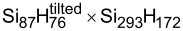 is given in the upper part of the figure. Calculated total CM, SSQC and CDCT lifetimes are reported in (a), (b) and (c), respectively, for the systems Si_87_H_76_ × Si_293_H_172_ and 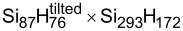, assuming *d* = 0.6 nm, for untilted and tilted systems (red and blue points, respectively). The size of the simulation box was 9.0 nm × 4.8 nm × 4.8 nm. The system Si_87_H_76_ × Si_87_H_76_ is depicted in the bottom-left part of the figure. Calculated total CM lifetimes for the system Si_87_H_76_ × Si_87_H_76_ are reported in (d) by assuming a NC–NC separation ranging from 0.9 to 0.1 nm. The reference (cross-type points) denotes the total CM lifetimes calculated for the isolated system (Si_87_H_76_). The size of the simulation box was 9.0 nm × 4.8 nm × 4.8 nm.

To support the general validity of our results, we analyzed CM effects considering two different additional systems. The first one is obtained by assuming a different configuration of Si_87_H_76_ × Si_293_H_172_, where the Si_87_H_76_ is rotated around one of axis of symmetry. In this new setup, denoted as 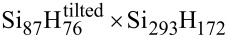, the NCs show a different reciprocal surface orientation that affects both wavefunction delocalization and spill-out parameters. The second one is obtained by placing in the same simulation box two identical Si-NCs, that is, Si_87_H_76_ × Si_87_H_76_, placed at a tunable separation (*d* = 0.9, 0.7, 0.5, 0.3, 0.1 nm). Calculated total CM, SSQC and CDCT lifetimes for the system 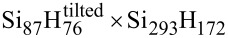 are depicted in Figure 4a–c. Simulated total CM lifetimes for the system Si_87_H_76_ × Si_87_H_76_ are reported in Figure 4d.

Despite the fact that the reciprocal NC orientation slightly affects both CDCT and SSQC lifetimes, we do not observe significant changes in CM dynamics from the Si_87_H_76_ × Si_293_H_172_ to the 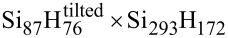 systems. Also, in this case, one-site processes dominate CM decay mechanisms and CDCT processes are faster than SSQC events.

Our conclusions do not change when we move from a system of differently coupled Si-NCs to a system of identically coupled Si-NCs. Also, in this case, NC interplay does not significantly affect sub-ps CM events that are dominated by the occurrence of one-site CM processes, that is, by processes that are only weakly influenced by NC–NC interaction. As a result, only CM decay paths with a lifetime greater than 1 ps are influenced by NC interplay and are then pushed to lower lifetimes.

As a result of ab initio calculations based on the first-order perturbation theory (weak coupling scheme), which is the one-site the dominant CM decay process, after absorption of a single photon we have always the formation of Auger-affected multiexcitons localized in single NCs, even when systems of strongly coupled NCs are considered. A direct separation of e–h pairs onto space separated nanostructures by SSQC is therefore not compatible with our theoretical results. Therefore, in our opinion, more complicated dynamics, where for instance SSQC effects are assisted by exciton recycling mechanisms [31,41], must be hypothesized in order to explain results of [36].

## Conclusion

In this work, we have calculated CM lifetimes for systems of isolated and interacting Si-NCs. As a first step, we have considered four different, free-standing NCs (Si_35_H_36_, Si_87_H_76_, Si_147_H_100_ and Si_293_H_172_) with diameters (energy gaps) ranging from 1.3 nm (3.42 eV) to 2.4 nm (1.70 eV). Calculated CM lifetimes have been reported using both an absolute and a relative energy scale. Recorded trends have been interpreted in terms of two-particle, effective Coulomb matrix elements, |*M*_eff_(*E*_i_)|, and of the density of final states, 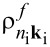
(E_i_) by dividing plots in two parts: a near CM energy threshold region (low energy region) and a far CM energy threshold region (high energy region). In this manner, we have proven that oscillations detected in the CM lifetimes plots at low energy are induced by fluctuations in the effective Coulomb matrix elements, while trends recorded at high energy are mainly connected with the monotonically increasing behavior of 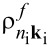
(E_i_). The role played by the screened part of the Coulomb potential (and by local fields) was then clarified.

The effects induced by NC interplay on CM dynamics have been investigated considering a system formed by two NCs placed in close proximity, that is, Si_87_H_76_ × Si_293_H_172_. One-site CM, SSQC and CDCT lifetimes have been quantified by first principles calculations and reported as a function of the energy of the initial carrier. The obtained results point out that one-site CM mechanisms always dominate over two-site CM processes and that the resulting lifetimes follow the hierarchy τ_one−site_ ≤ τ_CDCT_ ≤ τ_SSQC_. As a consequence, Auger affected multiexciton configurations are always formed in single NCs after absorption of high energy photons. A direct separation of e–h pairs in space-separated NCs is thus not compatible with our results. The role played by reciprocal NCs surface orientation has been investigated by rotating the Si_87_H_76_ system around one axis of symmetry. The obtained results indicated that although reciprocal NC orientation affects wavefunction delocalization (and thus the relevance of two-site CM processes, suggesting interaction between non-spherical NCs), it does not alter the hierarchy of lifetimes previously discussed. The same conclusions can be obtained when systems of identical, interacting, NCs are investigated. Moreover, in this case, the effects induced by NC interplay can only modify the efficiency of CM transitions with lifetimes higher than 1 ps.
